# Kickstand External Fixator for Immobilization Following Free Flap Plantar Calcaneal Reconstruction

**Published:** 2019-04-09

**Authors:** Swapnil D. Kachare, Bradley J. Vivace, Joshua T. Henderson, Milind Kachare, Christina Kapsalis, Jamie L. Fulfer, Joshua Choo, Bradon J. Wilhelmi

**Affiliations:** ^a^Division of Plastic and Reconstructive Surgery, Department of Surgery; ^b^School of Medicine, University of Louisville, Louisville, KY; ^c^Department of Surgery, Robert Wood Johnson Medical School, New Brunswick, NJ; ^d^Southern Illinois University School of Medicine, Carbondale, IL

**Keywords:** immobilization, external fixator, free flap, kickstand, heel reconstruction

## Abstract

**Objective:** Management of calcaneal wounds is challenging due to a paucity of tissue, complex local anatomy, and limited vascularity. These wounds are commonly associated with lower extremity fractures, which are often treated with external fixation. Free tissue transfers are frequently employed as a means for closure of plantar heel wounds; however, postoperative management can be challenging due to their dependent location. We sought to describe how simple modification of the external fixator can help relieve direct pressure, provide joint immobilization, and optimize accessibility necessary for flap survival. **Methods:** Three patients requiring autologous free tissue reconstruction of hindfoot defects were immobilized using an external fixator with a “kickstand” modification. Viability of the transferred tissue and the postoperative outcomes were assessed. **Results:** All free flaps survived with no associated complications. The “kickstand” modification was well tolerated with minimal discomfort. All 3 patients expressed satisfaction with early return to ambulation. **Conclusion:** An external fixator with a “kickstand” modification provides an essential function in maintaining the viability of the transferred tissue to plantar calcaneal wounds.

Multiple factors contribute to the failure of a healing wound.^1^ One factor that is commonly implicated is direct motion of the healing tissue. This is most true for free tissue transfers, especially those to the lower extremity, where persistent shear forces, dependent nature, positional pressure, and/or hasty return to weight-bearing can contribute to wound breakdown, infection, and loss of the flap. Therefore, it is not surprising that free flaps to the feet and ankles have a higher rate of failure than those of other anatomic sites.^2^

Calcaneal reconstruction is particularly vulnerable to shear forces due to the unique location and geometry of the calcaneus,^3^^,^^4^ therefore requiring the patient to be non–weight-bearing for at least 6 weeks to optimize tissue healing.^2^ During this time, patient positioning is extremely important to avoid compression of the flap base, minimize friction, and keep the area of the flap inset elevated.^2^^,^^5^ The use of adjuvant external fixators to immobilize the active joint and relieve direct pressure on the flap has previously been described.^6^^,^^7^ Several studies have illustrated the advantages of constructing “kickstands” created by adding additional rods to external fixators.^8^^-^10 Most recently, is 2013, Ting et al^11^ described techniques for an external fixator kickstand application in the setting of lower extremity soft tissue flaps.

Immobilization postreconstruction of the heel with a free flap is paramount.^2^ Many techniques exist for immobilization of feet or ankles following reconstruction with external fixation, but most of these require numerous transfixation pins, connectors, and rods.^2^^,^^6^^-^10 We present a simple immobilization technique used in 3 patients with lower extremity plantar heel injuries that were reconstructed with free soft tissue flaps.

## METHODS

### Patient 1

**Figure 1:**
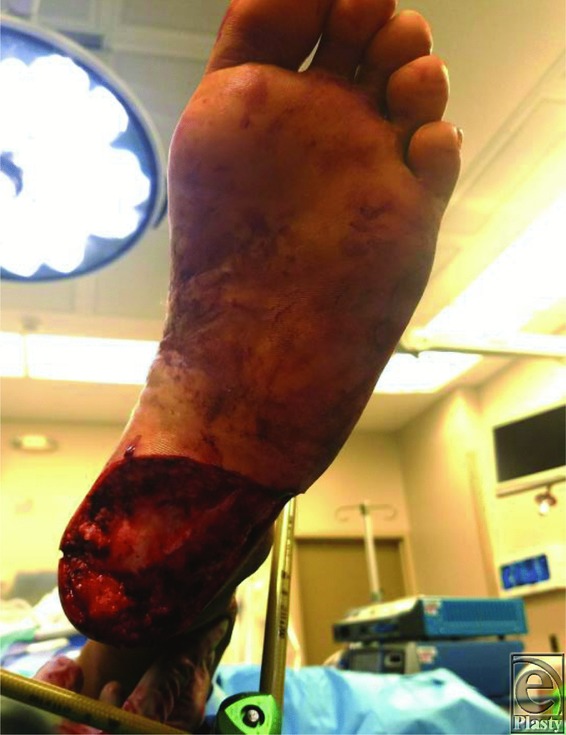
Heel wound postdebridement.

**Figure 2:**
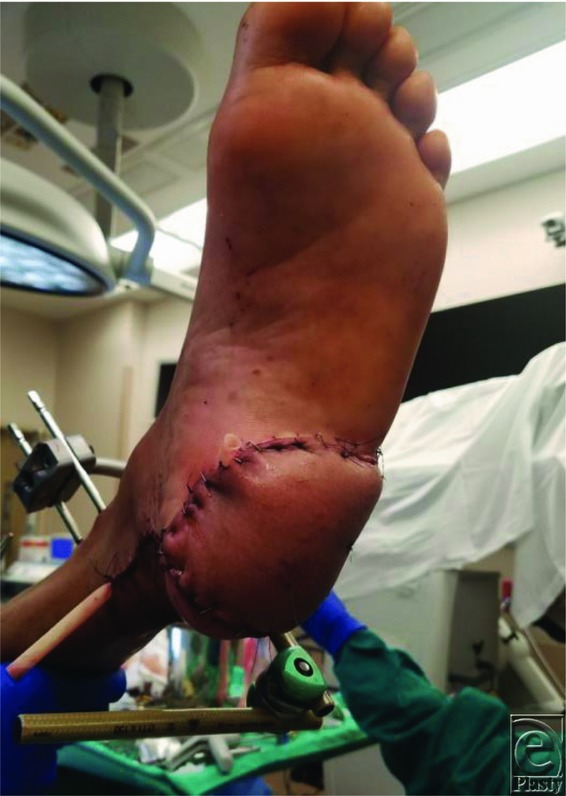
Heel wound postreconstruction.

**Figure 3:**
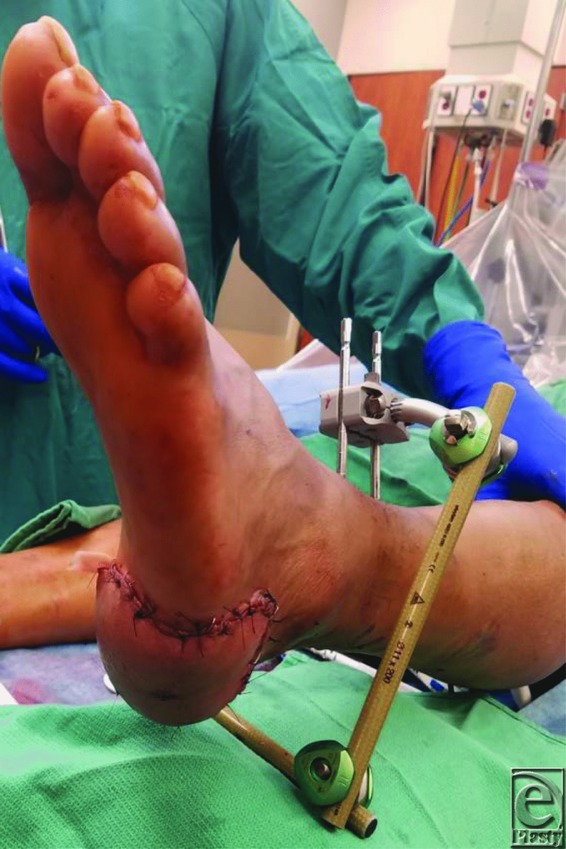
Completed kickstand postreconstruction.

A 29-year-old man was transferred to our facility following primary closure of an injury to the left heel sustained via hydraulic boom of a logging truck. This resulted in degloving of the skin overlying the posterior Achilles tendon and heel, creating a distal flap. Extensive debridement was performed for necrosis of the heel pad and skin over the calcaneal tendon (Fig 1). The resulting defect was reconstructed with a free latissimus dorsi myocutaneous flap (Fig 2). An external fixator with multiplane placement was employed for 18 days. Two additional rods and 2 connectors were used to create a “kickstand” (Fig 3). Repeat skin grafting was necessary with subsequent revision. The patient did well postdischarge, and the muscular flap remained viable.

### Patient 2

A 25-year-old man sustained a crush injury to the left heel by a bulldozer. Lower extremity fractures were repaired at an outside hospital, and he was transferred to our facility for reconstruction of the resulting soft tissue defect. The wound was repaired with a free latissimus dorsi myocutaneous flap and a split-thickness skin graft. The patient was placed in an external fixation for a total of 6 weeks. The external fixator was modified with additional bars to keep the heel elevated as seen in patient 1. The hospital course was complicated by a methicillin-resistant *Staphylococcus aureus* (MRSA) infection and hematoma at the injury site requiring evacuation. The patient did well after discharge, and the muscular flap remained viable.

### Patient 3

A 69-year-old man sustained third-degree frostbite to both feet. The patient underwent bilateral transmetatarsal amputation and required bilateral latissimus dorsi myocutaneous free flaps to cover the remaining defects. The patient was placed in an external fixator with kickstand modification on each leg as previously shown for 6 weeks. Revisional shortening of the metatarsals of the right foot was necessary secondary to partial flap necrosis. The patient did well after discharge, and the muscular flaps remained viable.

## RESULTS

A total of 4 latissimus dorsi myocutaneous free flaps were performed on 3 patients for trauma/burn wounds of the calcaneus. Each patient had external fixation with a kickstand modification to assist with postoperative flap care. All flaps survived, and each patient was ambulatory after reconstruction. The patients had minimal to no discomfort during the duration of immobilization, and they expressed high satisfaction.

## DISCUSSION

Management of lower extremity wounds following free flap reconstruction requires complex postoperative care, which is especially true for wounds of the heel. Use of an external fixator to assist with flap elevation provides a reliable method to minimize shear forces and pressure on the flap.^2^ There is a modest amount of literature available involving techniques for immobilization in patients who have undergone flap coverage of cutaneous heel injuries; however, much of the literature has focused on immobilization for cross-leg pedicle flaps.^12^^-^18 We describe a simple modification of the external fixator with a kickstand for the postoperative management of free flaps to plantar calcaneal wounds.

The use of external fixation for ankle injuries requiring soft tissue reconstruction was described by Mears,^19^ who discussed soft tissue reconstruction using soleus muscle transfer and split-thickness skin grafting. This approach utilizes either a quadrilateral frame or a triangular frame attached to transfixation pins and overhead suspension with traction.^19^ The benefits for external fixation were based on providing a stable fixation with easy accessibility for treating soft tissue injuries.^7^^,^^9^ Buford and Trzeciak^7^ described the use of a Hoffmann external fixation system for immobilization of the ankle following free flap reconstruction in 3 patients. They emphasized easy flap access and monitoring, elimination of foul-smelling dressings, and prevention of compression on the flap as benefits to this system.^7^ Our system provides similar benefits for plantar calcaneal wound, while keeping the heel and foot in an elevated position in relation to the remainder of the lower extremity.

The SALSAstand is similar to the kickstand we propose but with a different configuration. The SALSAstand anchors one base of the kickstand to pins inserted into the distal tibia and an additional transfixation pin inserted through the central portion of the first metatarsal shaft that exits the central portion of the fifth metatarsal shaft.^20^ Roukis and colleagues^21^ also described the use of a “kickstand” for management of plantar heel wounds, keeping the foot in the most elevated position in relation to the extremity. Unlike our study, they described use of a pedicle flap and insertion of 2 additional pins within the foot.^21^ The use of transfixation pins in the foot has been described to minimize equinous contracture in this patient population^11^^,^^22^; however, use of these additional pins increases risk of soft tissue injury,^22^ vascular injury,^23^ and potential risk of pin site fractures.^24^ Our kickstand does not require additional transfixation pins through the foot and requires fewer rods to provide the elevation. This placement keeps the foot elevated while minimizing risks of additional pin placement^23^^,^^24^ as well as the cost of rods, connectors, and pins.^7^^,^^11^ In addition, it affords the foot an increased range of motion, while maintaining immobilization of the posterior aspect of the heel, which is a critical difference between our kickstand and other configurations.^20^^,^^21^ To prevent equine deformity in our patient population, we employed thermoplastic plantar platforms and elastic slings that were fashioned to the external fixator.^11^^,^^21^

Mere elevation of the foot is associated with increased vascularity to the heel,^25^ and the use of external fixators has been described for prevention of heel ulcers.^9^ Therefore, the use of kickstands for postoperative management of free flaps to the heel is the next logical step; it allows for foot elevation and circumferential access to the flap and minimizes discomfort for the patient.^10^ The simplicity with which our kickstand external fixator can be constructed makes its use practical for the surgeon when managing patients who undergo free flap reconstruction of plantar calcaneal wounds.
